# Beyond Dominant Symbionts: Low-Abundance Taxa Govern Microbial Network Topology in Sympatric Ticks

**DOI:** 10.1007/s00248-026-02842-y

**Published:** 2026-07-20

**Authors:** Francisco J. Márquez, Y. Perez-Llano, S. A. Sánchez-Carrión, M. De Rojas, A. Caruz

**Affiliations:** 1 Department of Animal Biology, Vegetal Biology and Ecology, Faculty of Experimental Sciences, Universidad de Jaén, 23071 Jaén, Spain; 2 Department of Microbiology and Parasitology, Faculty of Pharmacy, University of Sevilla, Profesor García González 2, 41012 Sevilla, Spain; 3 Department of Experimental Biology, Faculty of Experimental Sciences, Universidad de Jaén, 23071 Jaén, Spain

**Keywords:** Ticks, Ixodidae, Microbiome, 16S rRNA gene sequencing, Ecological networks, Rare microbiome, Andalusia

## Abstract

Ticks are obligate hematophagous arthropods and major vectors of diverse bacterial, parasitic, and viral pathogens. They host complex microbial communities that critically influence their biology, fitness, and interactions with pathogens. Using 16 S rRNA gene amplicon sequencing at the amplicon sequence variant (ASV) resolution combined with co-occurrence network analysis, we characterized the bacterial communities of questing adult ticks collected in the Cazorla, Segura y Las Villas Natural Park (Jaén, Spain). A total of 83 adult ticks (27 males and 56 females) representing six sympatric species, *Dermacentor marginatus* (*n* = 39), *Haemaphysalis punctata* (*n* = 9), *H. sulcata* (*n* = 15), *Hyalomma lusitanicum* (*n* = 7), *Ixodes ricinus* s. l. (*n* = 2), and *Rhipicephalus bursa* (*n* = 11), were analyzed. After stringent quality filtering, 3.77 million high-quality reads were recovered and resolved into 407 ASVs. Due to the inherent resolution limits of the V3–V4 region for species-level discrimination, taxonomic assignments were conservatively consolidated at the genus level (101 bacterial genera). Across all tick species, the bacteriome was heavily dominated by Pseudomonadota (98.6%), with species-specific differences primarily driven by variation in obligate symbionts. *Coxiella*-associated ASVs predominated in multiple tick species, whereas *H. lusitanicum* exhibited strong dominance by *Francisella* and secondary representation of *Candidatus Midichloria*. Alpha and beta diversity analyses revealed distinct compositional patterns shaped by host identity, with lower dispersion observed in *D. marginatus* and *R. bursa*, and greater variability among *Hyalomma* and *Haemaphysalis* individuals. Spearman-based co-occurrence network analysis indicated a highly cooperative and modular structure (> 98% positive correlations) across tick species. Notably, dominant endosymbionts (*Coxiella*, *Rickettsia*, and *Francisella*) occupied peripheral or weakly connected positions within the networks. In contrast, ecological connectivity was governed by a distinct set of low-abundance taxa, including *Roseomonas*, *Friedmanniella*, *Methylobacterium*, *Sphingomonas*, *Aureimonas*, *Conexibacter*, *Marmoricola*, *Mycobacterium*, and *Nocardioides*, which acted as central hubs bridging network modules, a topology robustly validated by an independent, composition-aware (SparCC) reanalysis within the *D. marginatus* cohort. These comparative findings demonstrate a functional decoupling between abundance and connectivity in tick microbiomes, highlighting how the “rare microbiome” can drive the topological organization and potential stability of microbial communities across sympatric host species.

**Supplementary Information:**

The online version contains supplementary material available at https://doi.org/10.1007/s00248-026-02842-y.

## Introduction

Ticks are obligate blood-feeding arthropods that transmit a wide range of pathogens worldwide [21]. Due to their biology, these organisms act as vectors for a greater diversity of pathogenic agents, including bacteria, parasites, and viruses, than most other arthropod groups, and frequently harbour multiple agents simultaneously. This capacity is partly related to their ability to maintain and tolerate diverse microbial assemblages of viral [68] or bacterial origin [70–72, 83]. While the epidemiology of many tick-borne pathogens has been extensively studied, increasing attention has been directed toward the diversity, distribution, and biological roles of bacterial symbionts associated with ticks, including both intracellular and gut-associated microorganisms.

Beyond their relevance to vector-borne disease, tick–microorganism interactions represent a complex biological system in which commensal, mutualistic, and pathogenic microbes coexist and interact with their host [17, 30]. Vector saliva contributes to pathogen transmission through specific molecular mechanisms [49, 66, 81], while pathogens can induce changes in tick gene expression [64] that influence vector survival and physiology [17]. Consequently, the transmission of pathogens is increasingly viewed not solely as a by-product of hematophagy but as part of a complex biological system involving interactions between ticks and their associated microbiota [64].

Pathogen transmission by ticks typically occurs across all developmental stages of the tick life cycle. However, there are notable exceptions; for instance, *Anaplasma phagocytophilum* cannot be efficiently transmitted vertically by colonized adults. This absence of transovarial transmission is well-documented in *Ixodes* species, which serve as its primary vectors [90]. In striking contrast, vertical transmission does occur in *Dermacentor albipictus*, whose single-host life cycle represents a distinct ecological niche where transovarial transmission is evolutionarily favored [6]. Consequently, to the best of our current knowledge, a vertebrate reservoir host remains essential for maintaining the endemic cycle of *A. phagocytophilum* in typical multi-host tick ecosystems. Ticks can also acquire microorganisms during blood feeding from infected vertebrate hosts and retain them across moulting events, a process known as transstadial transmission, thereby enabling their passage to subsequent hosts [69]. Co-feeding transmission further enables microbial exchange between ticks feeding on the same host individual [7, 67, 93, 100]. These biological characteristics make ticks both important reservoirs and vectors of diverse microorganisms affecting animals and humans [5, 88, 89].

Advances in high-throughput sequencing technologies have revealed that ticks harbour complex and dynamic microbial communities [11, 42]. These microbial assemblages are increasingly recognized as important components influencing host physiology, metabolism, immunity, and interactions with pathogens [28]. The recognition that multicellular organisms exist in association with diverse microbial communities has led to integrative conceptual frameworks such as the holobiont and hologenome ([31]; a; [78]). However, the structure, variability, and ecological organization of tick-associated microbiota remain incompletely characterized, particularly under natural ecological conditions and across sympatric tick species.

Experimental studies have shown that perturbations of the microbiota can influence tick biology and pathogen transmission. For instance, manipulating the microbiota can decrease reproductive fitness in ticks such as *Dermacentor andersoni* [24] and *Amblyomma americanum* [103]. Dysbiosis has also been reported to alter pathogen colonization and transmission dynamics: it reduces *Borrelia burgdorferi* colonization in *Ixodes scapularis* [62, 63] and increases transstadial transmission of *Babesia microti* in *Haemaphysalis longicornis* [94]. These observations highlight the relevance of microbial communities in vector biology [54, 55, 97], although the ecological organization and interaction structure of these communities in wild tick populations remain insufficiently understood.

Despite growing recognition of the importance of tick-associated microbiota, several knowledge gaps persist. Most studies have focused on individual tick species, laboratory colonies, or limited geographic settings, leaving the comparative ecological structure of microbiomes across sympatric species under natural conditions poorly resolved. In addition, while compositional analyses have described dominant symbionts and community diversity, fewer studies have examined the broader association patterns among microbial taxa within ticks, including their potential organization into structured networks. Understanding how microbial assemblages vary among co-distributed tick species and how these communities are organized at the level of taxonomic associations remains essential for advancing ecological interpretations of tick–microbiome systems [22, 33, 101].

In this study, we characterized the bacterial communities of adult questing ticks collected from the Cazorla, Segura y Las Villas Natural Park in southern Spain using 16 S rRNA gene amplicon sequencing at amplicon sequence variant (ASV) resolution. We compared microbial composition and diversity across multiple sympatric tick species and examined patterns of taxonomic association using co-occurrence network analysis. The aim of this work was to describe the structure, variability, and association patterns of bacterial communities in wild tick populations under natural ecological conditions.

## Materials and Methods

### Tick Samples

**Fig. 1:**
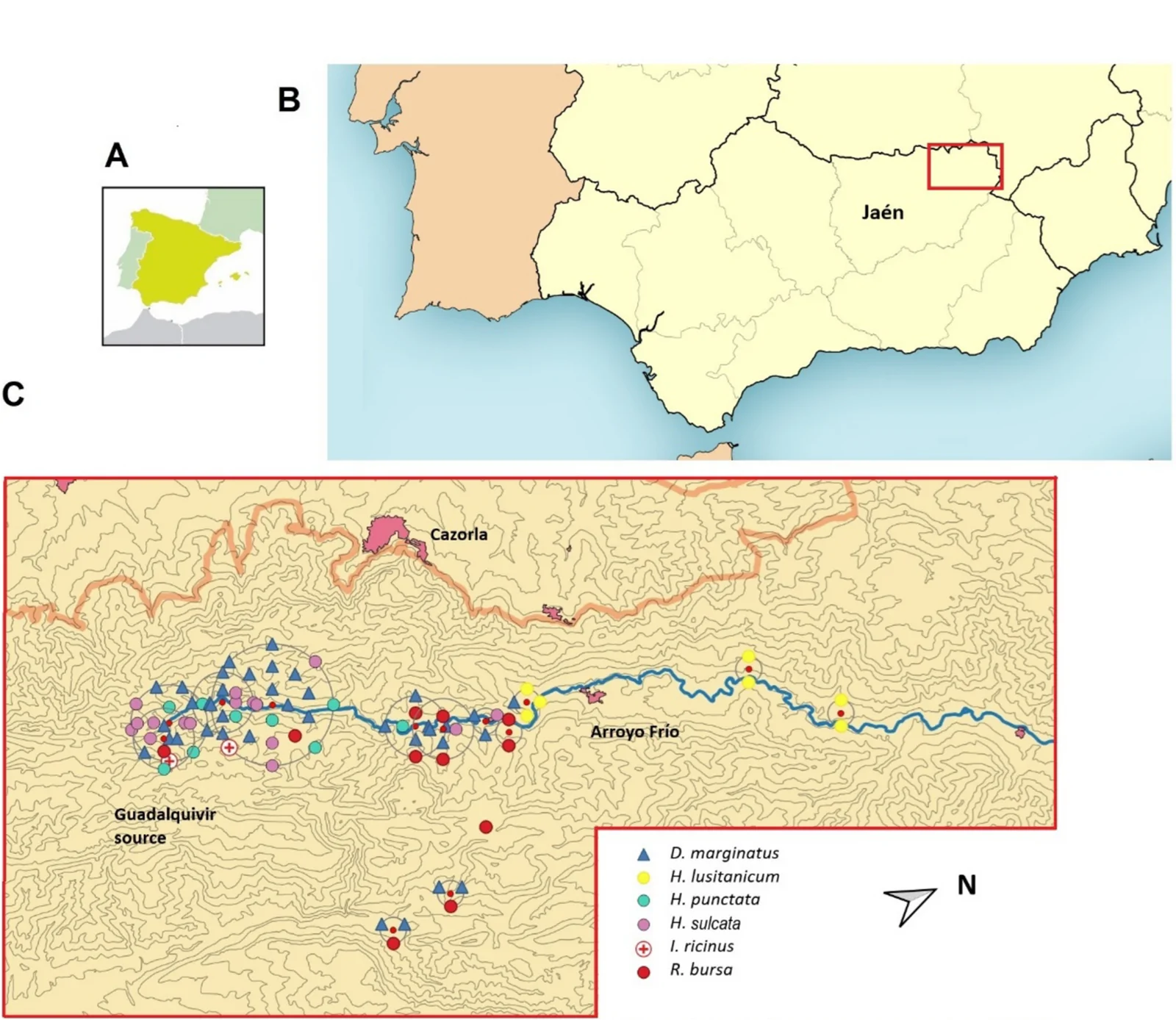
Study area. (**A**) Iberian Peninsula. (**B**) Cazorla, Segura y Las Villas Natural Park. (**C**) Tick collection sites and species distribution. Most samples originated from shrublands near the Guadalquivir River source and its mid-mountain course

Questing ticks were collected from vegetation in the Cazorla, Segura y Las Villas Natural Park (Jaén, Spain) using the flagging technique ([26, 27] (Fig. 1). Sampling was conducted during the peak period of adult tick activity (spring 2022 to spring 2023). From the collected material, 83 active adult specimens were selected for downstream molecular analysis based on their vitality and morphological integrity upon arrival at the laboratory.

Ticks were identified to species level and sex using standard morphological taxonomic keys [38]. Identification was based on diagnostic external characters observed under stereomicroscopy.

### DNA Extraction

DNA extraction was performed under strict aseptic conditions to minimize external contamination. Prior to extraction, ticks were surface-disinfected to reduce cuticular microbial DNA by immersion in 1% commercial bleach solution for 30 s, followed by three rinses of 1 min each in sterile distilled water [9].

Each tick was processed individually using sterile, single-use instruments. Specimens were placed in sterile Petri dishes and sectioned longitudinally to facilitate efficient tissue lysis. Total genomic DNA was extracted using the DNeasy Blood and Tissue Kit (Macherey-Nagel, Düren, Germany) according to the manufacturer’s instructions. All extractions were carried out in a dedicated clean workspace, and extraction blanks containing no tick material were included as negative controls to monitor potential contamination. DNA extraction, PCR preparation, and amplification steps were conducted in physically separated laboratory areas.

DNA concentration was quantified using a Qubit 3.0 fluorometer (Thermo Fisher Scientific) with the dsDNA High Sensitivity assay. DNA integrity and fragment size distribution were assessed by capillary electrophoresis using a Fragment Analyzer (AATI) with the Genomic DNA 50 kb kit. Only samples yielding DNA of sufficient concentration and quality were retained for subsequent 16 S rRNA gene amplification and sequencing.

### Library Preparation and Amplicon Sequencing

For the amplicon-based microbiome profiling, a total of 83 individual DNA samples were selected for 16 S rRNA gene amplification based on Next-Generation Sequencing (NGS). We prepared amplicon libraries targeting the V3–V4 variable region of the 16 S rRNA gene at the IPBLN Genomics Facility (CSIC, Granada, Spain) using a two-step PCR strategy according to the standard Illumina metagenomic library preparation protocol [48].

For the first PCR, 15 ng of genomic DNA per sample were amplified in triplicate 10 µL reactions using Nextera-adapted primers V3V4fw (5´CCTACGGGNBGCASCAG 3´) and V3V4rev (5’ GACTACNVGGGTATCTAATCC 3’) [91] at a final concentration of 0.2 µM. Amplification was performed using KAPA HiFi HotStart ReadyMix DNA polymerase (Roche Diagnostics, West Sussex, UK). To reduce amplification of host mitochondrial and plastid DNA, peptide nucleic acid (PNA) clamps were included in the reactions [52]. PCR cycling conditions consisted of an initial denaturation at 95 °C for 3 min, followed by 25 cycles of 95 °C for 30 s, 75 °C for 10 s, 55 °C for 30 s, and 72 °C for 30 s, with a final extension at 72 °C for 5 min. Triplicate reactions were pooled, and successful amplification was confirmed by agarose gel electrophoresis. Negative controls (extraction blanks and PCR non-template controls) were processed in parallel through all steps and consistently showed no visible bands or amplification artifacts on the agarose gels, confirming the absence of detectable background contamination prior to library pooling.

Amplicons were purified using the NucleoMag^®^ NGS Clean-up and Size Select Kit (Macherey-Nagel, Düren, Germany). In a second PCR step, dual combinatorial indices and Illumina sequencing adapters were added using the Nextera XT v2 Index Kit (Illumina, San Diego, CA, USA). This amplification consisted of an initial denaturation at 95 °C for 3 min, followed by 8 cycles of 95 °C for 30 s, 55 °C for 30 s, and 72 °C for 30 s, and a final extension at 72 °C for 5 min. Indexed amplicons were again purified using the NucleoMag^®^ NGS Clean-up and Size Select Kit and verified by agarose gel electrophoresis.

Purified libraries were quantified using a Qubit fluorometer (Thermo Fisher Scientific), pooled in equimolar concentrations, and assessed for fragment size distribution using a Bioanalyzer HS DNA chip. The pooled library was diluted and denatured according to Illumina guidelines. Sequencing was performed on an Illumina MiSeq platform using the MiSeq Reagent Kit v3 (600-cycle) with paired-end 2 × 300 bp chemistry.

### Sequence Processing and Microbiome Data Analysis

Raw paired-end reads were obtained in FASTQ format and processed using the DADA2 pipeline [19] implemented in the R environment (Posit Team [77]; [80]). Initial quality filtering and adapter trimming were performed using the *filterAndTrim* function, applying a maximum expected error threshold of 2.5 per read. No fixed truncation length was applied in order to preserve sequence overlap for downstream merging.

Filtered reads were dereplicated and subjected to amplicon sequence variant (ASV) inference using the dada algorithm with pseudo-pooling and self-consistent error learning (parameters err = NULL, pool = “pseudo”, selfConsist = TRUE). Forward and reverse reads were merged using a minimum overlap of 5 bp, and sequences failing to merge were discarded. Chimeric sequences were identified and removed using removeBimeraDenovo (DADA2), followed by an additional chimera screening step using the *FindChimeras* function in the DECIPHER package [96]. The resulting non-chimeric sequences constituted the final ASV dataset.

An ASV abundance table was constructed from the processed reads. ASVs representing less than 0.01% of total reads within a sample were removed to reduce potential spurious or low-confidence taxa. Taxonomic classification of ASV representative sequences was performed using the Naïve Bayesian classifier implemented in DADA2 against the SILVA reference database (version 138.2) [79, 99]. Representative sequences were additionally compared against the GenBank database using BLAST for confirmation of taxonomic assignments when necessary.

Downstream analyses were conducted using the phyloseq package in R [59]. Alpha diversity was estimated using observed richness, Shannon diversity index, inverse Simpson index, and Pielou’s evenness. Differences in alpha diversity among tick species were evaluated using the non-parametric Kruskal–Wallis test, followed by Dunn’s post hoc pairwise comparisons with Bonferroni correction. Tick species with insufficient field representation (i.e., fewer than three individuals per cohort) were excluded from these downstream inferential statistics to avoid underpowered statistical modeling and to ensure that differential abundance patterns were driven by robust, representative cohorts; descriptive data for these underrepresented cohorts were maintained solely for baseline qualitative comparison. Additionally, to evaluate potential intra-species variations driven by host sex, non-parametric Wilcoxon rank-sum tests were implemented to compare Shannon diversity scores between male and female ticks within species cohorts meeting a minimum threshold of ≥ 4 individuals per sex group.

Beta diversity was assessed by calculating Bray–Curtis dissimilarities based on ASV abundances. Prior to significance testing, homogeneity of multivariate dispersions (PERMDISP) was verified using the *betadisper* function. Variations in community composition among tick species were then evaluated via permutational multivariate analysis of variance (PERMANOVA) using the *adonis2* function; both analyses were implemented within the vegan R package (Oksanen et al., 2025). Pairwise post-hoc comparisons across tick species were subsequently resolved using the *pairwise.adonis2* function from the pairwiseAdonis package (v0.4.1; [58]). To ensure that these macro-comparative patterns were not confounded by host sex, independent stratified PERMANOVA models (999 permutations) were additionally executed based on the same Bray-Curtis distance matrices to formally test for structural and membership differences between males and females within the sex-stratified species cohorts. Lastly, community ordination patterns were visualized via Non-Metric Multidimensional Scaling (NMDS).

Core and shared genera among the investigated tick species were identified using the microbiome R package [51]. Core taxa were defined using a dual-threshold approach: a relative abundance ≥ 0.01% and a prevalence ≥ 40% within each group. The 0.01 abundance threshold was applied as a quality filter to eliminate low-frequency background noise and potential reagent contamination [10, 84]. Concurrently, the 40% prevalence threshold was selected to accommodate the high inter-individual and environmental microbiome variability typical of wild arthropod vectors, ensuring that context-dependent core symbionts were not artificially obscured by overly restrictive criteria [3, 82].

To establish an empirically justified prevalence filter for network construction and minimize the impact of transient or low-confidence taxa, a custom network sensitivity analysis was implemented. The dataset was partitioned by tick species. For each cohort, the relative prevalence of each ASV was calculated from the presence/absence matrix (defined by a detection threshold of > 0 reads) as the ratio of positive samples to the total sample size per species. ASV retention was systematically monitored across a strictness gradient of prevalence thresholds ranging from 5% to 30%. The final 20% threshold was selected by identifying structural plateaus and optimization inflection points where background noise was minimized while preserving a robust core matrix for downstream topological modeling. The resulting decay profiles were visualized using the *ggplot2* package [95].

### Microbial Co-occurrence Network Analysis

To investigate the taxonomic association patterns and structural organization of tick-associated bacterial communities, a dual network-modeling approach was implemented. First, a species-wide comparative network analysis was performed across all five investigated tick species using Spearman’s rank correlations. Second, a composition-aware network was constructed using the Sparse Correlations for Compositional Data (SparCC) algorithm to control for the mathematical artifacts inherent to high-throughput sequencing datasets [41]. All computational workflows, statistical evaluations, and network visualizations across this study were executed using R version 4.6.0 [80] within the RStudio integrated development environment version 2025.09.02 (Posit Team [77]). Specific network architectures, topological metrics, and graphical renderings were implemented using the specialized R packages *Hmisc* [44], *NetCoMi* [74], *igraph* [25], *ggraph* [73], and *qgraph* [37].

#### Comparative Cross-Species Networks (Spearman Rank Correlation)

Microbial co-occurrence networks were constructed separately for each of the five tick species. To limit the contribution of low-frequency taxa and mitigate spurious correlations associated with zero-inflated compositional data, the Amplicon Sequence Variant (ASV) abundance matrix was filtered by prevalence. The primary networks retained only taxa present in at least 20% of the samples within each species (Prevalence Threshold, PT = 0.2).

Pairwise associations among filtered ASVs were calculated using Spearman’s rank correlation via the *Hmisc* package. Correlation coefficients were adjusted for multiple testing using the Benjamini–Hochberg False Discovery Rate (FDR) approach. To assess network stability and filter out spurious correlations, co-occurrence networks were constructed and evaluated independently under two distinct FDR control levels, retaining edges only when |ρ| ≥ 0.65 and utilizing adjusted *p*-value thresholds of *p* < 0.05 and *p* < 0.01. The resulting adjacency matrices were modeled as undirected weighted networks using the *igraph* package. Global network properties, including the total number of nodes and edges, modularity, average clustering coefficient, and network diameter, were calculated for each species. At the node level, topological roles were evaluated through within-module degree (Z_i_) and participation coefficient (P_i_) to classify taxa according to their network connectivity.

To resolve the topological roles of individual ASVs beyond global network descriptors, each node was assigned a position in the Guimerà–Amaral functional-cartography space defined by its within-module degree (Zi; a Z-score of intramodular connectivity) and its participation coefficient (Pi); the evenness of an ASV’s edges across modules), and classified as a peripheral node (Zi ≤ 2.5, Pi ≤ 0.62), a connector (Zi ≤ 2.5, Pi > 0.62), a module hub (Zi > 2.5, Pi ≤ 0.62), or a network hub (Zi > 2.5, Pi > 0.62) following the thresholds of Guimerà and Nunes Amaral [43]. Nodes were categorized into four topological roles: peripherals, connectors, module hubs, or network hubs. Network visualization was performed using the *ggraph* package.

#### Composition-Aware Network Validation (SparCC)

To account for compositionality biases and the closure effect driven by dominant endosymbionts, an independent network analysis was performed using the SparCC algorithm. Due to the sample size requirements of this method to ensure algorithmic convergence, stable correlation estimations, and the prevention of false positives, SparCC modeling was exclusively applied to species cohorts with larger sample size (*N* ≥ 30 individuals).

The SparCC network was constructed at the genus level using the *NetCoMi* package. Empirical *p*-values were calculated through a bootstrap approach with 1,000 iterations and adjusted using the FDR method. A conservative threshold was applied to isolate the most reliable biological interactions, retaining only statistically significant correlations (adjusted *p* < 0.05 or *p* < 0.01) with an absolute correlation coefficient |r| ≥ 0.65 (positive edges: *r* ≥ 0.65; negative edges: *r*≤ −0.65) [1, 2]. Global and node-level topological metrics, including total nodes and edges, network diameter, modularity, average degree, weighted degree (strength), clustering coefficient, and overall network density, were computed using *igraph*, and the final composition-aware architecture was visualized using the *qgraph* package. Therefore, SparCC was used as a composition-aware validation of the best-powered species-specific network, corresponding to *D. marginatus*, whereas network inferences for the remaining tick species were based primarily on the Spearman correlation framework and should be interpreted comparatively within that methodological context.

## Results

The analyzed sample included 27 males and 56 females. Specimens belonged to six species: *Dermacentor marginatus* (*n* = 39), *Haemaphysalis punctata* (*n* = 9), *Haemaphysalis sulcata* (*n* = 15), *Hyalomma lusitanicum* (*n* = 7), *Ixodes ricinus sensu lato* (*n* = 2), and *Rhipicephalus bursa* (*n* = 11) (Table 1).

**Table 1:** Demographic composition of collected ticks. The table shows the total count per species and the breakdown between males and females. *Ixodes ricinus* (*N* = 2) was included for descriptive baseline purposes only and was excluded from all downstream comparative statistics and network analyses due to sample size limitations

Species	Females	Males	Total
*D. marginatus*	29	10	39
*H. lusitanicum*	6	1	7
*H. punctata*	4	5	9
*H. sulcata*	10	5	15
*I. ricinus*	2	0	2
*R. bursa*	5	6	11
Total	56	27	83

High-throughput 16 S rRNA gene sequencing generated a comprehensive dataset that enabled comparative evaluation of microbiome composition, diversity, and taxonomic association patterns across sympatric tick species.

### Microbiome Composition and Dominant Taxa

High-throughput sequencing generated a total of 3,771,178 high-quality non-chimeric reads, with an average of 45,435.9 sequences per sample (SD = 13,621). The generated 16 S rRNA amplicons exhibited a sequence length ranging from 401 to 428 base pairs (bp), with an average length of 414.85 ± 11.02 bp, confirming the successful and specific amplification of the targeted V3–V4 variable region. An ASV abundance table was constructed from these processed reads. To eliminate potential spurious or low-confidence taxa, ASVs representing less than 0.01% of total reads within a sample were strictly removed. Taxonomic classification of the remaining ASV representative sequences was performed using the Naïve Bayesian classifier implemented in DADA2 against the SILVA reference database (version 138.2; [79, 99] and additionally validated against the GenBank database using BLAST when necessary.

To avoid computing artefactual ecological interactions, Amplicon Sequence Variants (ASVs) lacking a confident classification at the genus level were systematically discarded prior to downstream analyses. Furthermore, downstream statistical and ecological modeling was conservatively consolidated at the genus level rather than the species level. This choice was justified by the inherent technical limitations of short-read V3–V4 amplicons, which lack sufficient phylogenetic resolution to reliably differentiate closely related species within dominant, highly conserved tick-associated lineages (e.g., *Coxiella*, *Francisella*, and *Rickettsia*), thereby preventing potential taxonomic misassignment. After applying these stringent quality and taxonomic curation filters, the final dataset comprised 407 robust ASVs aggregated into 101 bacterial genera, distributed across 7 phyla and 54 families (Supplementary Table S1).

Across the entire dataset, the tick bacteriome was strongly dominated by the phylum Pseudomonadota (98.64% of total reads), followed by Actinobacteriota (0.86%), Bacteroidota (0.35%), and Bacillota (0.12%). Other phyla, including Deinococcota, Planctomycetota, and Abditibacteriota, were detected at very low relative abundance (< 0.01%). Detailed relative abundance profiles across all evaluated tick species from the order down to the genus level are comprehensively compiled in Supplementary Table S2.

**Fig. 2:**
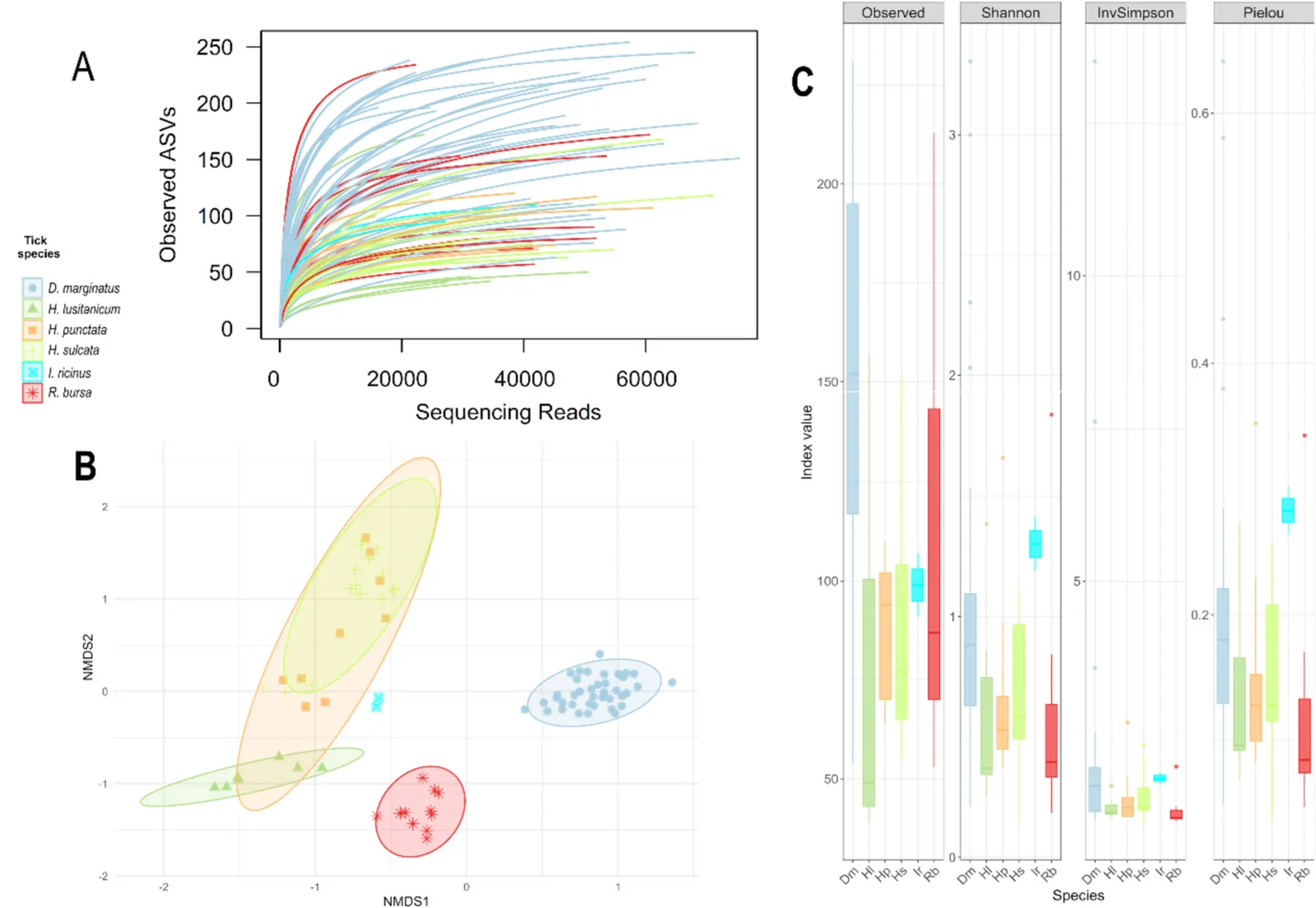
(**A**) Rarefaction curves of the bacterial microbiota across the investigated tick species. The curves illustrate the number of observed Amplicon Sequence Variants (ASVs) as a function of the sequencing depth (number of reads) per individual sample. Individual lines are color-coded by tick species according to the legend. The asymptotic plateau observed across all curves confirms that the sequencing effort achieved sufficient depth to capture the total microbial richness within the sample cohort. (**B**) NMDS analysis based on Bray-Curtis distances for Mediterranean tick bacteriomes. Ordination plot showing the separation of microbial communities by host species. Points are shaped and coloured by species. The low stress value (Stress = 0.186) indicates an excellent representation of the high-dimensional data in two dimensions. Differences in community structure were statistically significant according to PERMANOVA (F = 12.4, *p* < 0.001), with post-hoc pairwise comparisons indicating that the *H. punctata* and *H. sulcata* bacteriomes are significantly distinct from both *D. marginatus* and *R. bursa*. (**C**) Effective abundance, Shannon diversity index (H’) and inverse simpson index (1/D) were calculated for alpha diversity, as well as Pielou’s evenness index

To verify whether the sequencing depth was sufficient to adequately represent the tick-associated bacterial communities, rarefaction analysis was performed across all samples. The rarefaction curves for all tick species exhibited a clear asymptotic behavior, approaching a plateau at their respective sequencing limits (Fig. 2A). Importantly, despite the high dominance of primary endosymbionts (such as *Francisella*-like endosymbionts [FLEs] or *Coxiella*-like endosymbionts [CLEs]), that characteristically overwhelm tick sequencing datasets, this strong saturation effect demonstrates that the sequencing coverage was highly robust. The depth achieved per sample was sufficient to thoroughly capture the vast majority of the microbial richness, including the lower-abundance bacterial fraction, ensuring that further sequencing would not significantly alter the observed community structures.

A Non-metric Multidimensional Scaling (NMDS) analysis based on Bray-Curtis dissimilarity was performed (stress value of 0.186). The NMDS plot revealed clear and significant clustering driven by tick species (Fig. 2B). Three species, *D. marginatus*, *R. bursa*, and *I. ricinus*, formed highly distinct, non-overlapping clusters. The tight grouping of samples within each of these ellipses suggests that their respective microbial communities are both unique and relatively homogeneous (low intra-species variation). For species *I. ricinus*, the small sample size (*n* = 2) precluded the calculation of a confidence ellipse, though its two points also cluster distinctly from all other groups. In stark contrast, the confidence ellipses for species *H. lusitanicum*, *H. punctata*, and *H. sulcata* showed a high degree of overlap and greater sample dispersion. This pattern indicates that the microbial compositions of these three species are less distinct from one another and exhibit higher intra-species variability compared to *D. marginatus*, *R. bursa*, and *I. ricinus*. Effective abundance, Shannon Diversity Index (H’) and Inverse Simpson Index (1/D) were calculated for alpha diversity, as well as Pielou’s Evenness Index (Fig. 2C). Marked differences in community composition (beta diversity) were observed among tick species.

To address potential sex-specific biases within the tick microbiome, comparative diversity analyses were performed across the four cohorts with adequate statistical power. PERMANOVA models based on Bray-Curtis distances demonstrated that tick sex was not a significant driver of overall microbial community structure (beta diversity) in any of the examined species, showing no significant variance replication in *D. marginatus* (*R*^*2*^ = 0.060, *p* = 0.058), *R. bursa* (*R*^*2*^ = 0.170, *p* = 0.125), or *H. punctata* (*R*^*2*^ = 0.337, *p* = 0.071).

Regarding alpha diversity, non-parametric tests confirmed that bacterial richness and evenness (Shannon index) remained statistically equivalent between males and females in *R. bursa* (*p* = 0.1775), *H. punctata* (*p* = 0.9048), and *D. marginatus* (*p* = 0.0506). Conversely, *Haemaphysalis sulcata* exhibited a distinct pattern; while its overall community structure (beta diversity) remained uniform and unaffected by sex (*R*^*2*^ = 0.027, *p* = 0.493), a statistically significant divergence was detected in its alpha diversity (*p* = 0.0193), indicating that sex-driven variations in *H. sulcata* are confined to changes in specific taxon richness rather than a complete rearrangement of the core microbiome architecture. Nevertheless, this significant alpha-diversity finding warrants a cautious interpretation given the relatively small sample size within this cohort. Furthermore, these within-species sex comparisons were evaluated as independent, exploratory pairwise tests, and no cross-species correction for multiple testing was applied.

**Fig. 3:**
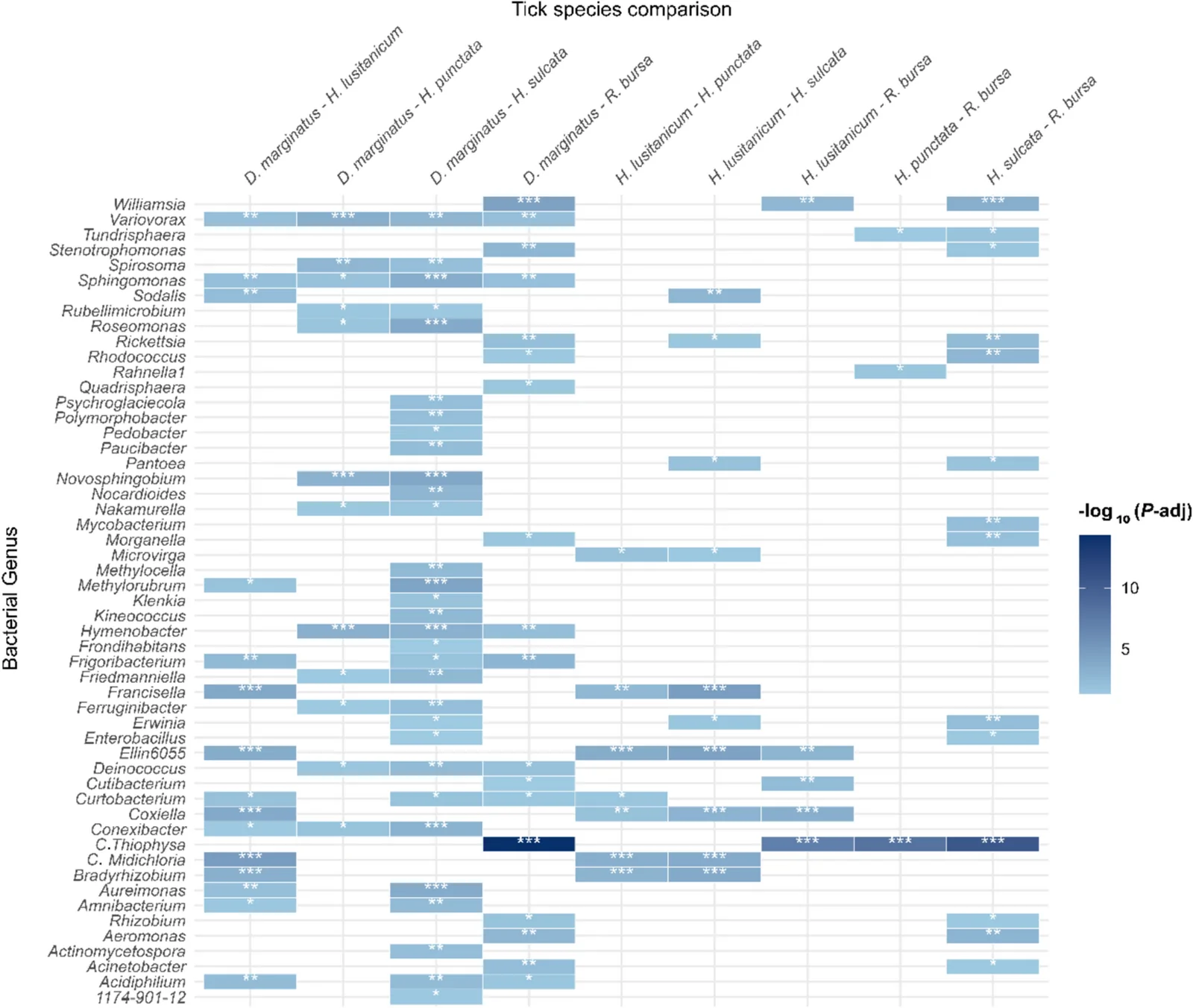
Pairwise comparison of dominant bacterial genera across mediterranean tick species. This heatmap illustrates bacterial genera showing statistically significant variations in relative abundance among the compared tick species. The color scale represents the -log_10_-transformed Bonferroni-adjusted p-values derived from Dunn’s post hoc tests, where darker blue shades indicate higher statistical significance. Differences in genus-level abundance were initially evaluated using a non-parametric Kruskal–Wallis test. Significant pairwise associations are further highlighted with asterisks within the cells (*: *p* < 0.05; **: *p* < 0.01; ***: *p* < 0.001)

Differences in bacterial abundance across well-represented tick species were determined using the Kruskal–Wallis test. To ensure statistical integrity and avoid underpowered modeling due to sample size asymmetry, *Ixodes ricinus* was intentionally excluded from all inferential comparative analyses. This approach led to the identification of 53 bacterial genera exhibiting statistically significant variations among the remaining five tick species. To pinpoint specific pairwise differences, we subsequently employed Dunn’s post hoc test with Bonferroni correction, and the resulting adjusted *p*-values (-log_10_ transformed) were visualized via a high-resolution heatmap (Fig. 3).

The updated heatmap reveals that the most extensive microbial profile divergences were driven by comparisons involving *D. marginatus*, particularly against *H. sulcata* and *H. lusitanicum*, displaying a massive block of highly significant variations (*p* < 0.001). Notably, *Ca. Thiophysa* emerged as one of the most prominent markers of host-species divergence, showing highly significant differential abundance (*p* < 0.001) across almost all pairwise comparisons, except for those involving *H. lusitanicum* vs. *H. punctata* and *D. marginatus* vs. *H. punctata*.

Furthermore, traditional endosymbionts and associated taxa acted as key drivers of this inter-species variation. *Ca. Midichloria* and *Bradyrhizobium* displayed highly significant differences (*p* < 0.001) exclusively in comparisons involving *D. marginatus* vs. *H. lusitanicum* and *H. lusitanicum* vs. *H. sulcata*/*R. bursa*. Similarly, *Francisella* exhibited highly significant differential abundance (*p* < 0.001) when comparing *H. lusitanicum* against *D. marginatus*, *H. punctata*, and *H. sulcata*. Other genera, such as *Sphingomonas*, *Ellin6055*, and *Variovorax*, also contributed robustly to the characterization of these distinct vector-specific microbial signatures.

**Fig. 4:**
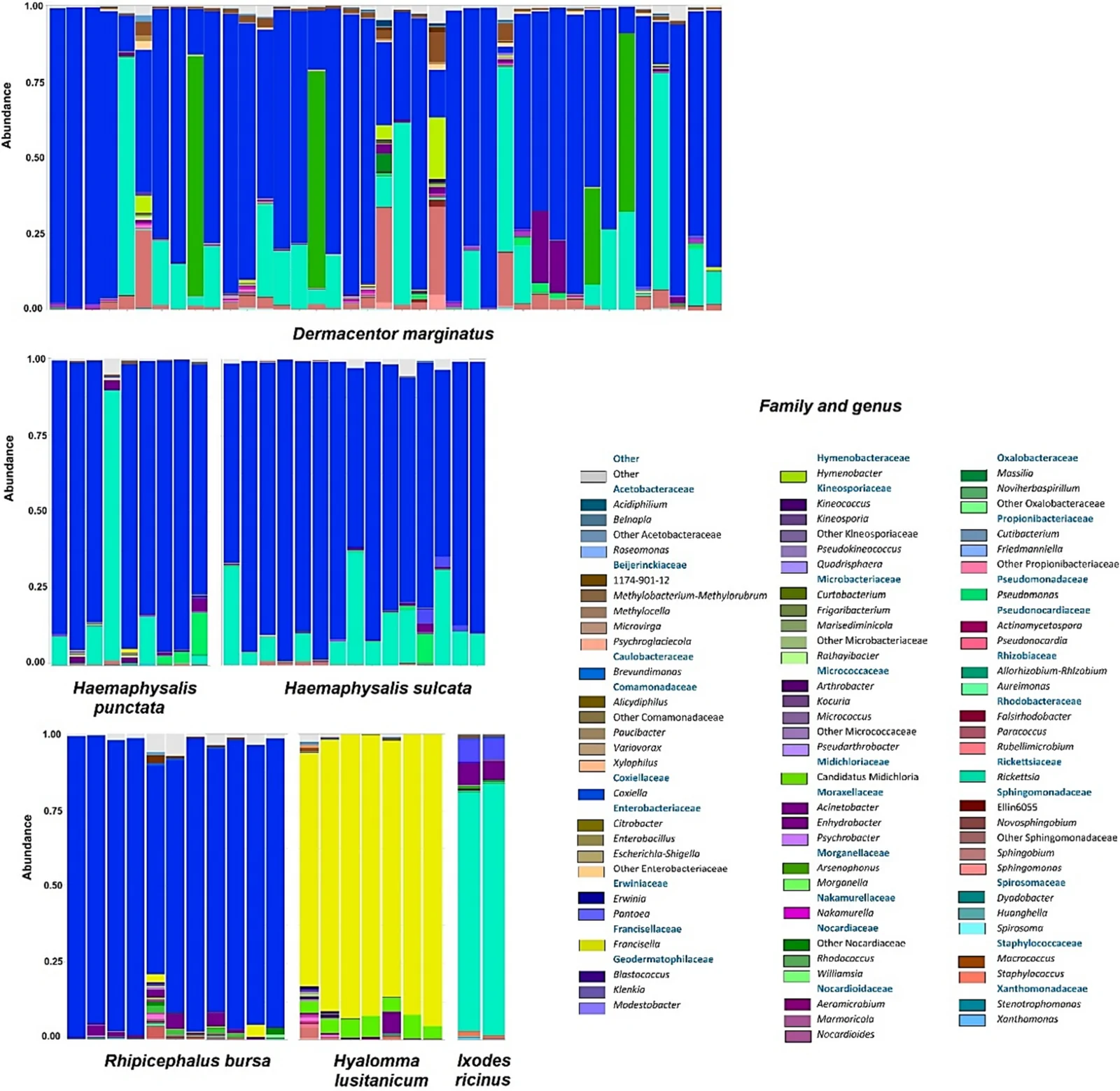
Relative abundance of bacterial families across individual tick samples. Each vertical bar represents the bacteriome composition of a single tick individual, grouped by host species. Taxa are color-coded at the family level. To enhance clarity, only the most frequent families (mean relative abundance > 1%) are shown individually; all remaining taxa are grouped into the “others” category. Taxonomic assignments were performed against the SILVA database (v.138)

As reflected in the stacked bar plot (Fig. 4), which presents the 20 most frequent bacterial families for each studied tick species, a diverse array of both pathogenic and non-pathogenic bacterial genera was identified within all tick species.

**Fig. 5:**
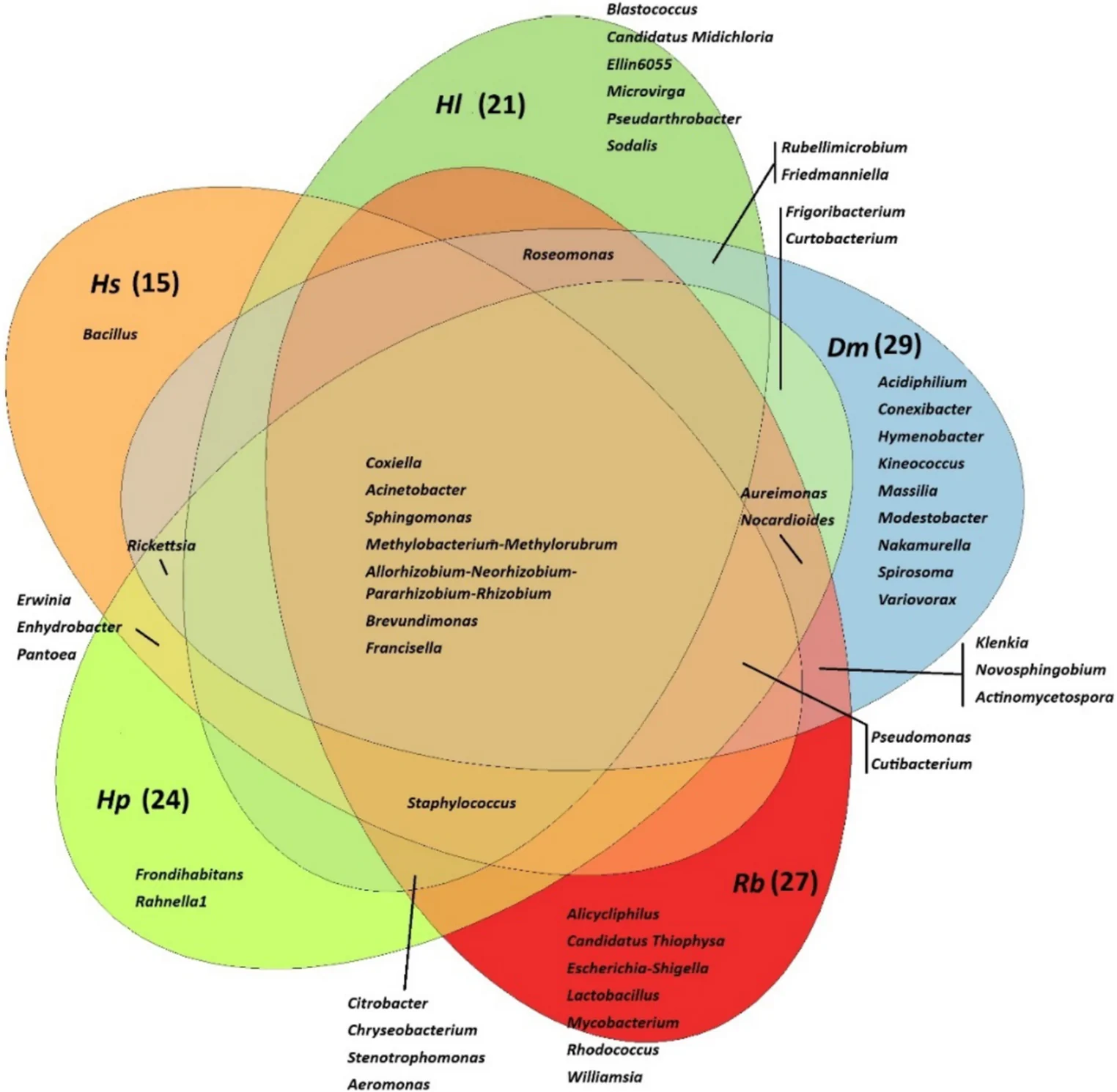
Distribution of shared and operationally exclusive bacterial genera across five mediterranean tick species. The Venn diagram illustrates the structural overlap of the resident microbiota among *Dermacentor marginatus*, *Hyalomma lusitanicum*, *H. punctata*, *Haemaphysalis sulcata* and *Rhipicephalus bursa*, computed using a strict 40% prevalence threshold (prevalence filter = 0.4) to filter out transient, low-frequency environmental taxa. The total number of analyzed specimens for each cohort is indicated in parentheses. Central intersections represent the shared core microbiome maintained at high prevalence across all hosts, whereas peripheral segments highlight bacterial genera whose stable colonization (≥ 40% prevalence) is operationally restricted to a single tick species

To characterize the distribution and structural overlap of the stable microbial components across the five well-represented tick species, a Venn diagram was constructed using a strict 40% prevalence threshold (prevalence filter = 0.4) (Fig. 5). This operational filter was selected to eliminate transient, low-frequency environmental taxa and sequencing noise, which typically distort raw comparative profiles and obscure host-specific signatures. Under this framework, ‘exclusive’ taxa do not imply absolute biological absence in other tick species at trace levels, but rather indicate that their stable, high-prevalence colonization (≥ 40% of individuals within a cohort) is strictly restricted to a single tick host.

The analysis revealed distinct core and accessory profiles, with *D. marginatus* (29 genera) and *R. bursa* (27 genera) exhibiting the highest core richness, followed by *H. punctata* (24), *H. lusitanicum* (21), and *H. sulcata* (15). A shared core microbiota consisting of seven highly prevalent genera (*Coxiella*, *Acinetobacter*, *Sphingomonas*, *Methylobacterium-Methylorubrum*, *Allorhizobium-Neorhizobium-Pararhizobium-Rhizobium*, *Brevundimonas*, and *Francisella*) was consistently maintained across all five tick species above the 40% threshold.

The analysis of the tick bacteriome revealed a community structure characterized by strong dominance of a limited number of taxa and clear host-associated patterns. Distinct dominance profiles were observed across tick species, with most communities showing high relative abundance of Coxiella-associated ASVs. This Coxiella-dominated pattern was present in the majority of species, where a single symbiont accounted for most of the bacterial community. *D. marginatus* exhibited a microbiome characterized by high relative abundance of Coxiella_ASV1, with Coxiella_ASV9 present at lower but consistent abundance. In contrast, *R. bursa* was predominantly associated with a different variant, Coxiella_ASV2. The two *Haemaphysalis* species showed concurrent high relative abundance of Coxiella_ASV3 and Coxiella_ASV4. In two species, *Coxiella*-associated ASVs were not dominant; instead, different taxa represented the major components of the bacterial community [53, 65]. The bacteriome of *I. ricinus* was characterized by high relative abundance of Rickettsia_ASV11. Similarly, *H. lusitanicum* showed strong dominance of Francisella_ASV7, with C. Midichloria_ASV15 consistently present at lower abundance. Overall, these patterns indicate species-associated differences in the identity of dominant ASVs, with community structure largely reflecting variation in dominant symbiont composition rather than functional guild organization or inferred competitive exclusion. Secondary taxa were present at lower relative abundance and varied across species, contributing to differences in community composition but not altering the overall dominance structure.

### Network Structure and Topological Roles of the Tick Microbiome

**Fig. 6:**
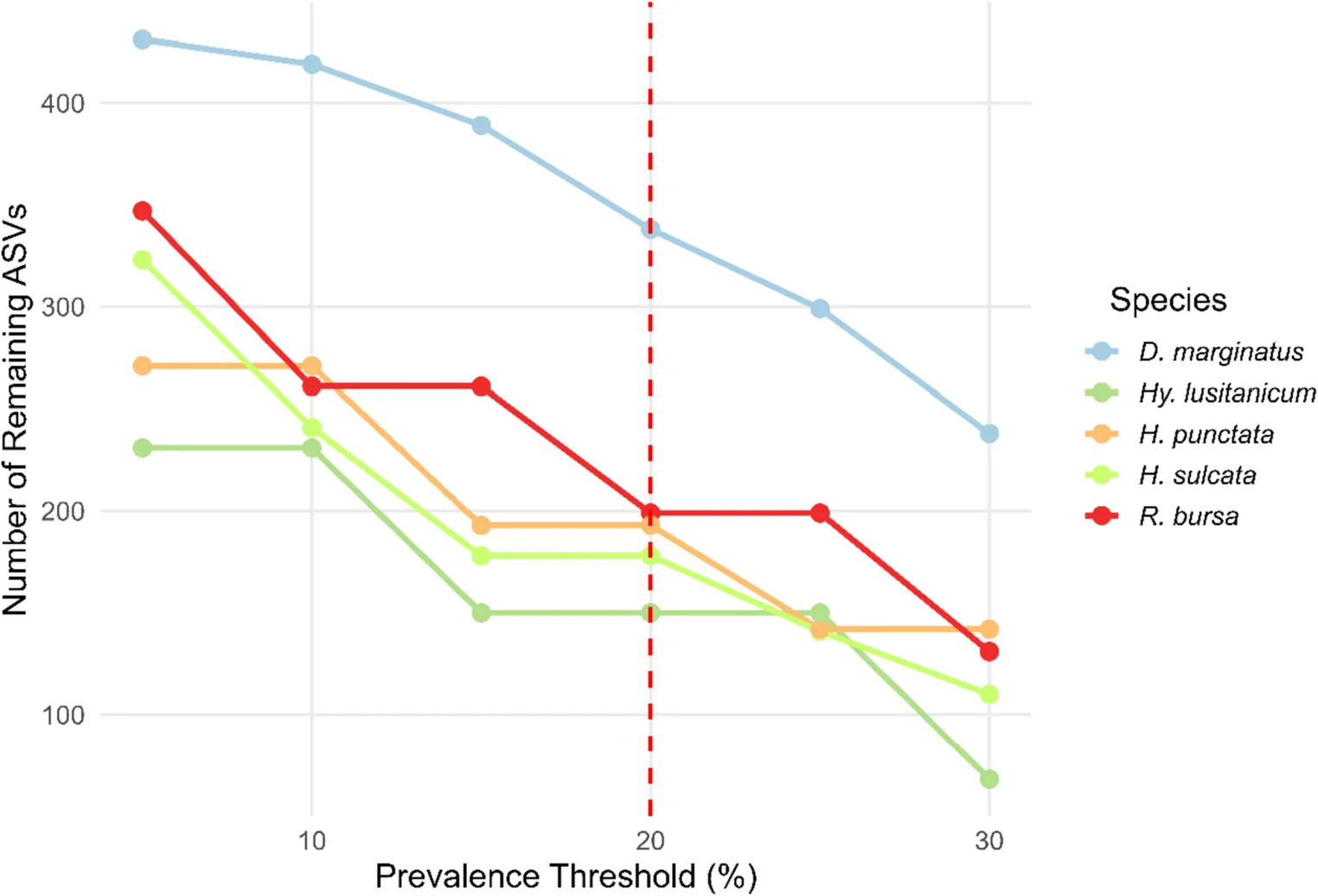
Prevalence threshold (PT) sensitivity analysis for microbial co-occurrence networks. The plot illustrates the decay in the number of remaining Amplicon Sequence Variants (ASVs) across the investigated tick species as the filtration PT increases from 5% to 30%. The vertical red dashed line highlights the selected 20% PT. Distinct horizontal plateaus (e.g., between 15% and 20% for *Haemaphysalis* spp. and *Hy. lusitanicum*, and between 20% and 25% for *R. bursa*) justify the mathematical stability of the 20% cutoff, ensuring the removal of low-frequency transient taxa while preserving robust community structures for downstream topological analysis

Before network construction, a sensitivity analysis was executed to evaluate the impact of data filtering across tick species (Fig. 6). For *H. sulcata*, *H. punctata*, and *Hy. lusitanicum*, the number of retained ASVs remained strictly constant between the 15% and 20% thresholds, demonstrating an ultra-stable core microbiota. Furthermore, *R. bursa* reached a distinct structural plateau precisely at the 20% mark. For the highly diverse *D. marginatus*, the 20% threshold successfully cleared low-frequency background noise while preserving a robust matrix of 340 mathematical nodes, validating the 20% threshold for downstream topological analyses.

#### Global Network Topology

Co-occurrence networks were constructed at a prevalence threshold (PT) of 0.2 using Spearman rank correlations [|ρ| ≥ 0.65, adjusted False Discovery Rate (FDR) *p* < 0.05 or *p* < 0.01] (Table 2).

**Table 2:** Topological features of microbial networks prevalence threshold (PT = 0.2), |ρ| ≥ 0.65, and FDR < 0.05 or < 0.01

Metric (FDR: <0.05/<0.01)	D. marginatus	H. lusitanicum	H. punctata	H. sulcata	*R*. bursa
Nodes	199/199	127/102	135/108	131/107	164/130
Edges	623/623	2123/1616	321/162	604/314	1499/578
Modularity	0.581/0.570	0.125/0.091	0.869/0.908	0.619/0.669	0.342/0.539
Clustering coeff.	0.491/0.491	0.857/0.957	0.804/0.795	0.604/0.692	0.686/0.689
Diameter	10/10	7/5	8/4	11/12	11/10

Network topology varied substantially among the evaluated tick species, though all consistently demonstrated a modular organization. Modularity gradients revealed a distinct biological spectrum ranging from highly interconnected, open networks to structures segregated into independent blocks (Table 2). Furthermore, pronounced inter-species variations were observed regarding number of nodes, number of edges, clustering coefficients, and topological diameters.

*Dermacentor marginatus* exhibited the largest network in terms of node complexity, maintaining a remarkably stable structure across both FDR thresholds, complemented by intermediate levels of modularity and clustering. Interestingly, *Dermacentor marginatus* exhibited high topological stability to FDR thresholding, retaining its exact network size (199 nodes and 623 edges) and macro-topological properties regardless of the False Discovery Rate (FDR) stringency (Table 2). Far from being a methodological artifact, this mathematical immunity to FDR thresholding (from < 0.05 to < 0.01) highlights the extreme statistical robustness of its underlying microbial interactions.

Similarly, *R. bursa* displayed a robust and complex topology, defined by a substantial number of nodes and connections coupled with an intermediate clustering coefficient. Occupying an intermediate space, *H. sulcata* exhibited moderate dimensions combined with a relatively high modularity and an extended topological diameter, suggesting a locally cohesive yet broadly distributed network. Finally, *H. punctata* was characterized by a markedly fragmented architecture; despite presenting a highly reduced number of interactions across its nodes, it recorded the highest modularity in the study, confirming a co-occurrence pattern strictly dominated by isolated yet internally tightly-knit bacterial modules.

In sharp contrast, *H. lusitanicum* was characterized by having, by a wide margin, the highest interaction density (edges) despite possessing a smaller number of nodes. This extreme cohesion was reflected in the highest clustering coefficient across the study and a near-zero modularity, defining a highly compact, open microbial matrix with a reduced topological diameter.

#### Cooperative Nature of Critical Network Connectivity

Analysis of the sign distribution of correlations associated with highly connected nodes revealed a consistent pattern across species: most associations involving hub ASVs were positive. Both inter-modular connectors and nodes with high within-module connectivity showed a strong predominance of positive correlations, with positive links exceeding 98% on average. For example, hub-associated ASVs in *R. bursa* (99.8%) and *D. marginatus* (99.7%) were almost exclusively linked through positive correlations, while *H. punctata*, despite showing the highest proportion of negative associations among species, still maintained a high prevalence of positive links (96.8%).

These patterns indicate that highly connected taxa tend to co-vary positively with other members of the community and are positioned within regions of the network characterized by predominantly positive associations. While correlation-based networks do not demonstrate direct ecological interactions, the observed sign distribution is consistent with a community structure in which dominant and highly connected taxa are associated with broadly concordant abundance patterns rather than strong antagonistic relationships.

#### Node Roles and Highly Connected ASVs

The utilization of Amplicon Sequence Variants (ASVs) enabled the differentiation of distinct sequence-level taxonomic units and the characterization of their relative topological positions within the species-specific co-occurrence networks. Topological analysis based on among-module connectivity (P_i_) and within-module degree (Z_i_) identified a total of 44 ASVs acting as critical connector nodes (Z_i_ < 2.5 and P_i_ > 0.62), which facilitate communication across different modules rather than exercising a strong within-module dominance.

**Fig. 7:**
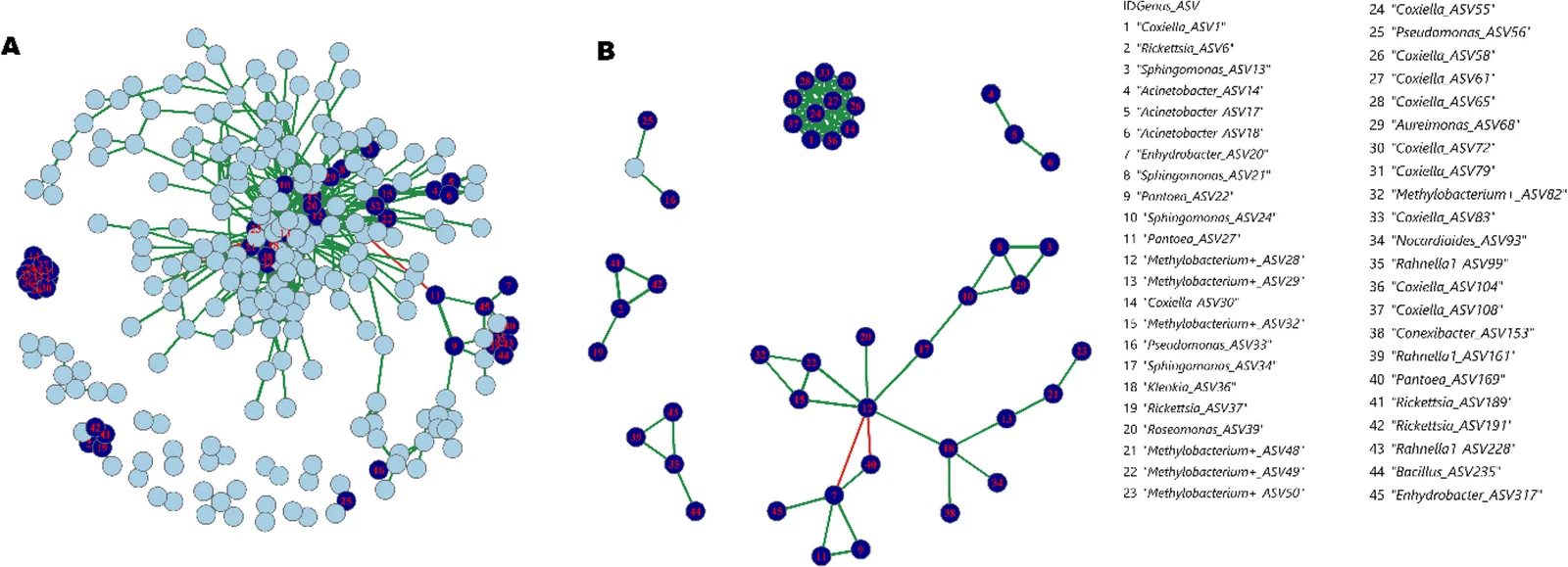
Comparative co-occurrence network analysis of the *Dermacentor* bacterial microbiota. (**A**) Naive network constructed using Spearman rank correlation (|r| ≥ 0.65, FDR *p* ≤ 0.05), displaying a hyper-connected structure caused by compositional bias and the closure effect driven by dominant endosymbionts. (**B**) Composition-aware network constructed using the SparCC algorithm (|r| ≥ 0.65, Bootstrap FDR *p* ≤ 0.05), which controls for compositionality, revealing a sparse and robust biological backbone. Dark blue nodes represent taxa consistently identified across both network architectures, highlighting the resilient core microbiome preserved after filtering out spurious correlation artifacts (light blue nodes)

**Fig. 8:**
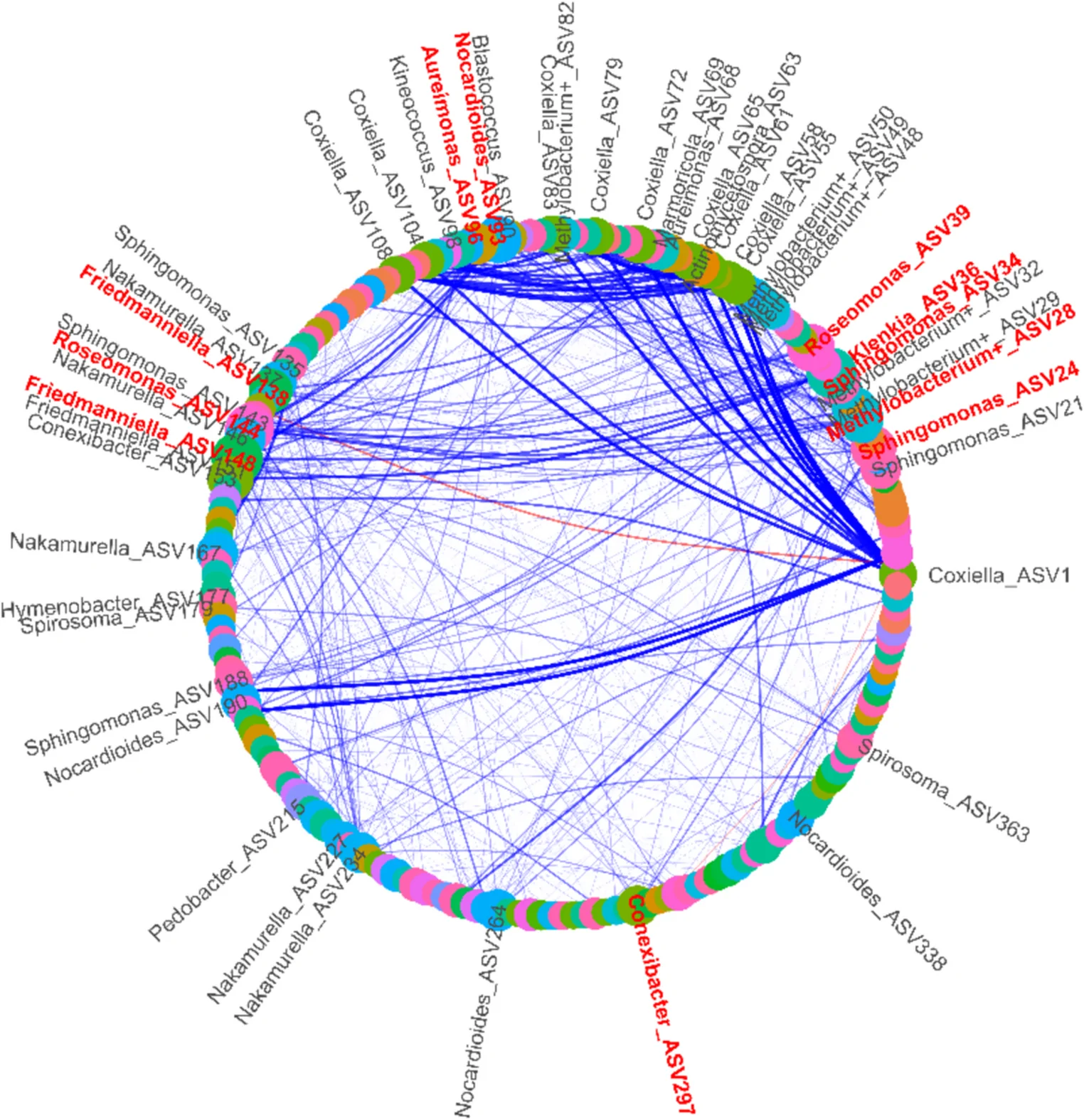
Circular co-occurrence networks (Arc Plot) for the microbial communities of *D. marginatus*. Each node represents an individual ASV, where the size of the circle is proportional to the topological Strength metric, reflecting the weighted sum of the node’s connections. Node color indicates taxonomic assignment at the genus level. The edges (links) between nodes represent significant Spearman correlations, computed under the same conditions as detailed in Fig. 7. To avoid visual overlapping and highlight the most relevant taxa in the network, labels (Genus_ASV) were filtered based on their structural importance: nodes identified as Critical Hubs are highlighted in bold red typography, whereas nodes corresponding to the top 25% with the highest connection strength (75th percentile) are shown in standard black text. Labels for the remaining peripheral nodes have been omitted to preserve visual clarity

In the *Dermacentor marginatus* network, the topological structure was anchored by a distinct group of critical hubs and highly connected peripheral-stabilizing nodes (Figs. 7 and 8). The critical hubs (highlighted in bold red) heavily featured actinobacterial and proteobacterial genera, most notably *Roseomonas* (ASV39, ASV144), *Nocardioides* (ASV93), *Friedmanniella* (ASV138, ASV148), and *Sphingomonas* (ASV24, ASV34). Additionally, the top 25% of nodes with the highest connection strength (displayed in standard black text) included prominent representatives of *Nakamurella* (ASV146, ASV227) and *Nocardioides* (ASV264, ASV338), alongside a massively connected *Coxiella* node (ASV1) that acted as a major interaction anchor within the community. The SparCC composition-aware validation supports the robustness of the inferred *D. marginatus* network backbone, because a subset of taxa and associations remained detectable after correcting for compositional effects. However, because SparCC was not applied to the lower-powered species cohorts, cross-species conclusions regarding network topology remain primarily supported by the Spearman-based comparative networks.

**Fig. 9:**
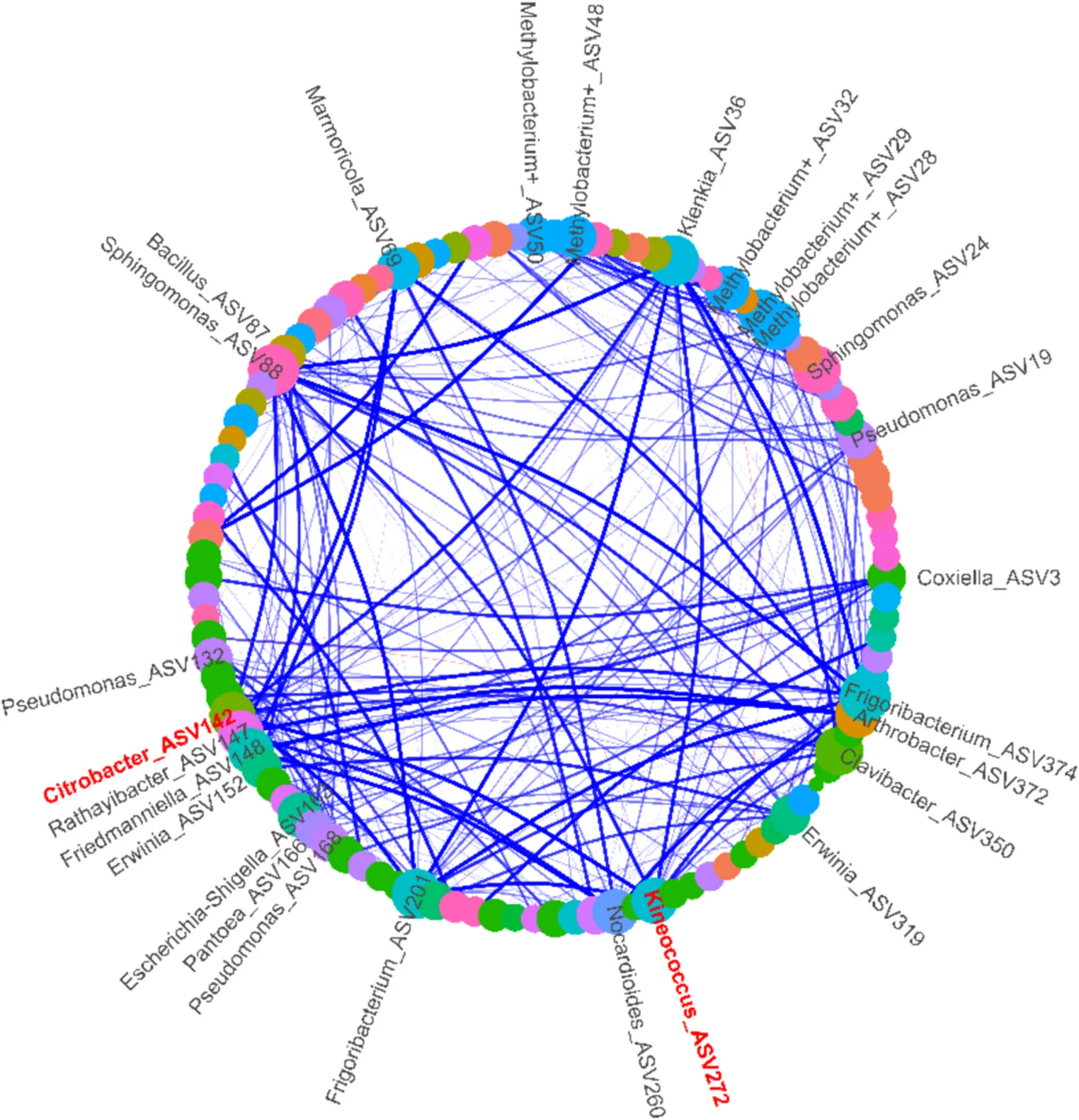
Circular co-occurrence networks (Arc Plot) for the microbial communities of *H. sulcata*. Nodes identified as critical hubs are highlighted in bold red typography, whereas nodes corresponding to the top 25% with the highest connection strength (75th percentile) are shown in standard black text. Labels for the remaining peripheral nodes have been omitted to preserve visual clarity

In contrast to the multi-hub architecture observed in *D. marginatus*, *H. sulcata* exhibited a more centralized topological structure anchored by only two distinct critical hubs (highlighted in bold red): *Citrobacter* (ASV142) and *Kineococcus* (ASV272) (Fig. 9). Furthermore, its top 25% of nodes with the highest connection strength (displayed in standard black text) revealed a specialized structural backbone heavily dominated by specific actinobacterial genera, such as *Clavibacter* (ASV350), *Arthrobacter* (ASV372), *Frigoribacterium* (ASV201, ASV374), and *Rathayibacter* (ASV147), alongside multiple *Methylobacterium+* variants (ASV28, ASV29, ASV32, ASV50). Interestingly, mirroring the pattern described for *D. marginatus*, the *H. sulcata* network was also heavily anchored by a highly connected *Coxiella* node (ASV3), highlighting the conserved role of this dominant endosymbiont as a primary interaction framework across different tick hosts, despite variations in their taxonomic support network.

To validate the key topological nodes, we categorized the operational roles of specific ASVs based on their within-module degree (Z_i_) and participation coefficient (P_i_ across *R. bursa*, *D. marginatus*, and *H. sulcata* (Table 3; Figs. 8 and 9).

**Table 3:** Topological classification and operational roles of key bacterial ASVs based on network modularity metrics. For each host tick species (*R. bursa*, *D. marginatus*, and *H. sulcata*), individual ASVs were categorized according to their within-module degree (Z_i_) and participation coefficient (Pi). Shared connectors represent core taxa maintaining equivalent high-connectivity positions across multiple host species. Primary symbionts and species-specific drivers highlight operational network anchors restricted to a single host background. Modularity thresholds for topological roles follow standard network ecology frameworks, where connectors bridge distinct functional modules (P_i_ ≥ 0.62) and hubs stabilize localized sub-graphs (Z_i_ > 1.0). Complete taxonomic assignments and network visualizations corresponding to these metrics are displayed in Figs. 8 and 9

Tick Species	ASV ID	Taxon (Genus)	Within-module degree (Z_i_)	Participation coeff. (*P*_i_)	Ecological/Topological significance
Shared Connectors					
*R. bursa*	ASV34	*Sphingomonas*	−1.679	0.686	Universal Connector (Shared among all three species)
*D. marginatus*	ASV34	*Sphingomonas*	0.401	0.785	
*H. sulcata*	ASV34	*Sphingomonas*	−0.67	0.653	
*R. bursa*	ASV28	*Methylobacterium+*	0.597	0.684	Inter-species shared connector (*R. bursa/D. marginatus*)
*D. marginatus*	ASV28	*Methylobacterium+*	1.274	0.678	
*R. bursa*	ASV151	*Friedmanniella*	0.439	0.649	
*D. marginatus*	ASV151	*Friedmanniella*	0.924	0.643	
*R. bursa*	ASV210	*Pseudonocardia*	0.697	0.7	
*D. marginatus*	ASV210	*Pseudonocardia*	−0.887	0.625	
Primary Symbionts					
*R. bursa*	ASV5	*Coxiella*	−0.126	0.743	Primary endosymbiont acting as network bridge
*R. bursa*	ASV164	*Coxiella*	−0.488	0.722	
Species-Specific Drivers					
*R. bursa*	ASV36	*Klenkia*	0.559	0.714	High-connectivity module bridging taxon
*R. bursa*	ASV337	*Mycobacterium*	0.785	0.711	
*D. marginatus*	ASV264	*Nocardioides*	0.171	0.741	
*D. marginatus*	ASV39	*Roseomonas*	1.935	0.716	
*H. sulcata*	ASV62	*Methylobacterium+*	−0.228	0.656	Species-specific connector
*H. sulcata*	ASV103	*Staphylococcus*	−1.302	0.667	
*H. sulcata*	ASV298	*Acinetobacter*	−0.931	0.625	

In contrast to the peripheral placement of dominant symbionts, microbial network connectivity was strictly governed by a distinct set of low-abundance taxa acting as key topological anchors (Table 3). This approach revealed a highly conserved ecological framework across the studied vectors. Most notably, *Sphingomonas* ASV34 emerged as a universal connector, maintaining a consistently high participation coefficient across all three tick species, which demonstrates its cross-species role in bridging distinct microbial modules.

Concurrently, network modularity and local cohesion were heavily supported by a specialized suite of host-specific drivers. Specifically, *Roseomonas* and *Friedmanniella* acted as central facilitators stabilizing the *Dermacentor marginatus* network, while genera such as *Methylobacterium*, *Aureimonas*, *Conexibacter*, *Marmoricola*, *Mycobacterium*, and *Nocardioides* functioned as critical localized drivers within their respective tick cohorts.

Furthermore, a specialized inter-species backbone was identified linking *R. bursa* and *D. marginatus*, driven by shared module-bridging connectors such as *Methylobacterium+* ASV28, *Friedmanniella* ASV151, and *Pseudonocardia* ASV210.

In contrast to these shared structural elements, species-specific drivers highlighted distinct evolutionary adaptations in network stabilization. In *R. bursa*, the network core was heavily influenced by its primary endosymbionts; here, two distinct *Coxiella* variants (ASV5 and ASV164) functioned as critical network bridges, structurally supported by high-connectivity taxa like *Klenkia* ASV36.

Conversely, *D. marginatus* relied on unique high-connectivity drivers, such as *Roseomonas* ASV39 (which exhibited the highest within-module degree) and *Nocardioides* ASV264, both standing out as prominent featured hubs in the optimized layout (Fig. 8). Lastly, *H. sulcata* displayed a highly streamlined, specialized architecture governed by localized species-specific connectors, including *Staphylococcus* ASV103, *Methylobacterium+* ASV62, and *Acinetobacter* ASV298 (Fig. 9).

Collectively, these topological Z_i_-P_i_ metrics confirm that while peripheral ambient taxa fluctuate depending on the host background, Mediterranean tick microbiomes maintain a strict structural hierarchy governed by predictable universal connectors and primary endosymbiont anchors. Crucially, within the best-powered *D. marginatus* network, this robust cooperative architecture was fully reproduced and validated under an independent, composition-aware (SparCC) reanalysis, confirming that low-abundance taxa, rather than dominant endosymbionts, bridge the primary network modules in this representative cohort.

## Discussion

Ticks are well-known hosts for a diverse array of microorganisms, including commensals, endosymbionts, and pathogens [22, 47, 60] These microbes can profoundly influence their tick hosts, with effects ranging from detrimental to neutral or beneficial [75]. Beyond their immediate impact, they contribute to critical physiological processes in ticks, such as nutritional adaptation, growth, reproduction, resistance to environmental stressors, and immunity [12, 50, 75, 104]. Furthermore, ticks are competent vectors and transmitters of several important pathogens, including species from the phyla Spirochaetota (e.g., *Borrelia* spp.) and Pseudomonadota (e.g., *Anaplasma* spp., *Coxiella* spp., *Ehrlichia* spp., *Francisella* spp., and *Rickettsia* spp.) which are uniquely adapted for development within their tick vectors for at least part of their lifecycle and hold undeniable public health significance [29, 86]. Indeed, ticks offer a fascinating, albeit complex, model for investigating the composition, function, and ecological impact of their bacterial communities, thereby warranting increased research attention [11]. Understanding the relationships between tick-borne microorganisms and their symbiotic interactions is crucial for developing effective future strategies to control arthropod pests and mitigate vector-borne pathogen transmission.

Beyond the intricate network interactions, the baseline composition and diversity metrics offer fundamental insights into the microbial ecology of the studied ixodid ticks within the Sierra de Cazorla region. Our taxonomic profiling revealed a community structure frequently dominated by primary endosymbionts, showcasing a clear host-associated dichotomy. On one hand, Coxiella was highly prevalent in *Dermacentor*,* Haemaphysalis*, and *Rhipicephalus* [55, 76, 102]; on the other hand, *Hyalomma* species displayed a characteristic dominance of *Francisella*-like endosymbionts ([32]Fig. 4). This overarching pattern is widely documented in wild tick populations due to the essential, long-standing mutualistic role of these maternally inherited microbes, which supply vital B-vitamins and cofactor biosynthetic pathways lacking in the host’s strict blood meal [14, 35, 56].

Interestingly, the alpha diversity patterns indicated that while certain species maintain a high richness of commensal bacteria, others present significantly lowered diversity indices. This reduction is often a direct consequence of endosymbiont hyper-abundance, which effectively crowds out transient or minor commensal taxa. Furthermore, the beta diversity analyses demonstrated a clear compositional segregation among the bacteriomes of *D. marginatus*,* R. bursa*,* H. lusitanicum*,* H. punctata* and *H. sulcata*, despite their sympatric distribution and potential for co-feeding on shared wildlife hosts [20]. This distinct clustering highlights that environmental acquisition through the host’s life cycle is strictly filtered by species-specific physiological barriers and micro-ecological niches. Crucially, these foundational composition and diversity patterns lay the groundwork for our network outcomes; the high compositional divergence observed across tick hosts explains why the microbial core architectures are ultimately governed by species-specific drivers rather than a structurally conserved, universal network (Table 3).

These findings offer intriguing insights into host-microbiome eco-evolutionary dynamics. Based on the concept of phylosymbiosis [30], one might anticipate that taxonomically related ixodid ticks would maintain highly congruent microbial network structures. However, our results demonstrate a clear divergence; while a single universal connector (*Sphingomonas*) maintains cross-species integrity, the core architecture is governed by species-specific drivers unique to each tick host (Table 3). This lack of strict network conservation implies that individual host factors, such as specific physiological niches, life-history traits, or local environmental exposures, phylogenetic constraints in shaping the cooperative bottlenecks of the tick microbiome. Our characterization of the tick-associated bacteriome in the southeastern Iberian Peninsula, encompassing six cohabiting and frequently co-feeding species, represents a significant advance in understanding the microbial ecology of these vectors in a region with high tick diversity, where some of these species have notable epidemiological relevance [57].

### Tick Bacteriome: Diversity, Core Composition, and Symbiosis

Our characterisation of the tick-associated bacteriome reveals a predominance of Pseudomonadota, consistent with previous studies [32, 61, 76] The core bacteriome includes *Coxiella*,* Acinetobacter*,* Sphingomonas*, and *Francisella* [98], likely resulting from evolutionary and adaptive conservation [16, 76]. These results reinforce the notion of evolutionarily driven microbial associations [45, 92].

This is consistent with a recent meta-analysis of *Hyalomma* bacteriomes, primarily focusing on populations in the Middle East and North Africa, indicated that the genera *Acinetobacter*, *Bacillus*, *Flavobacterium*, *Francisella*, *Rickettsia*, *Staphylococcus*, *Pseudomonas*, and *Corynebacterium* are consistently identified across multiple studies [1, 4].

Our results indicate that *Candidatus Midichloria mitochondrii* is a highly frequent and dominant component of the microbiome specifically within H. lusitanicum, whereas it is present with very low, negligible significance in *R. bursa* and the remaining tick species analyzed here [55]. This pattern explains its classification as an operationally exclusive genus for *H. lusitanicum* under our strict 40% prevalence threshold (Fig. 5). However, when contextualized with broader literature, the distribution of this endosymbiont is known to extend across multiple hard tick species (Ixodidae) globally, including the primary vector of Lyme disease, *Ixodes ricinus* [8], as well as *Rhipicephalus bursa* [36] and *Hyalomma lusitanicum* [87]. Its distribution also extends to soft ticks (Argasidae), suggesting a widespread association with these arthropod vectors [18]. A defining characteristic of *C*. *M. mitochondrii* is its unique and variable intracellular localization; while some bacteria are found within the host cell cytoplasm, others exhibit a distinctive tropism for the mitochondria, localizing specifically in the intermembrane space between the inner and outer mitochondrial membranes [36, 85].

### Core Symbionts: Metabolic Roles and Host Specificity

The stable core is built upon obligate endosymbionts like *Coxiella*-like endosymbionts (CLEs) and *Francisella*-like endosymbionts (FLEs), which compensate for nutritional deficiencies in the blood meal [23, 35]. The high prevalence of FLEs in *H. lusitanicum* and CLEs across *Rhipicephalus*,* Dermacentor*, and *Haemaphysalis* reinforces patterns of host-genus specificity [1, 22, 34, 53, 103] and presumptive origin of these bacteria [13]. The microbial communities can vary significantly with the host and location, such as the high prevalence of *C. Midichloria* in *Ixodes* [85] and *Hyalomma* species [46]. The high prevalence of these specific endosymbionts underscores clear patterns of host-genus specificity. Metagenomic analysis reveals that *Midichloria* rescues the nutritional symbiosis in *H. marginatum* by providing the biotin pathways lost through *Francisella*’s pseudogenisation [15].

### Network Robustness and Ecological Strategies

To move beyond simple abundance metrics, we applied co-occurrence network analysis. The reliability of such networks is critically dependent on the filtering of spurious correlations. We validated our approach by maintaining a fixed prevalence threshold (PT = 0.2) and comparing network topologies under two FDR levels (Table 2). Lowering the FDR threshold to 0.01 significantly reduced network size in most species, such as *H. punctata* and *R. bursa*, while increasing or maintaining high modularity, confirming that this stricter filter successfully isolates the robust, structural backbone of the microbiome. Interestingly, *D. marginatus* exhibited exceptional stability, retaining its exact network size (199 nodes and 623 edges) regardless of the FDR stringency, which highlights the baseline resilience of its inferred microbial interactions.

The response to this topological filtering revealed divergent ecological strategies among the studied tick species (Table 3). *Dermacentor marginatus* exhibited a remarkably resilient architecture; its network stability relies on a combination of the universal facilitator *Sphingomonas* and specialized, host-adapted species-specific drivers, such as *Roseomonas*, to mediate global network flow even under stricter thresholds. In sharp contrast, the *R. bursa* network lost its local cohesion, retaining no unique species-specific drivers and relying solely on the primary symbiont *Coxiella* alongside *Sphingomonas* (Table 3). This suggests that its apparent connectivity is statistically vulnerable, a fragility consistent with a bacteriome heavily saturated by a vertically transmitted obligate symbiont characterized by low network connectivity, leaving the remaining commensal community without the specialized topological drivers necessary to build network resilience. Finally, *H. sulcata* displayed a pattern of balanced modular robustness. It maintains a highly integrated network supported by its own distinct drivers, such as *Klenkia* and *Acinetobacter*, which only become structurally apparent once background correlational noise is removed. These varying topological strategies (shared connectivity vs. local driver reliance) likely reflect host-specific evolutionary adaptations to distinct internal microenvironments.

A crucial insight from our topological analysis is the functional positioning of obligate symbionts. The microbial network architecture is defined by high modularity, confirming a highly compartmentalised ecosystem. Despite their overwhelming abundance, ASVs corresponding to *Coxiella* and *Rickettsia* were consistently classified as Peripheral nodes or simple Connectors, rather than formal Module Hubs (Z_i_ > 2.5). This topological segregation is hypothesized to confer resilience to the network [39, 43]. High prevalence and overwhelming dominance of specific *Francisella*-like or *Coxiella*-like symbionts in *Dermacentor*, *Haemaphysalis*, *Hyalomma* and *Rhipicephalus* species, may support the concept of one symbiont displacing others (niche substitution) to become the core dominant specialist [34]. By operating peripherally, the modules containing these essential functions are predicted to be insulated from competitive dynamics or cascading failures that may arise from perturbations affecting opportunistic bacteria. To further assess this topological independence, the network construction was specifically scrutinised to evaluate the role of the obligate endosymbionts *Coxiella* and *Francisella*. Contrary to being entirely excluded from the network, ASVs corresponding to both *Coxiella* and *Francisella* were consistently included but were exclusively classified as peripheral or simple connector nodes across all species and prevalence threshold considered. This topological finding confirms their low network connectivity from the community’s critical dynamics: their stable, vertical transmission and obligate nutrient provisioning roles appear independent of the interaction patterns exhibited by the environmental and resident commensal microbiota [14, 16, 35].

Furthermore, a significant biological divergence was observed in *H. lusitanicum*, where *Coxiella* was displaced to a residual load (low counts), suggesting a clear niche substitution by the dominant *Francisella* and highlighting a major difference in symbiont ecology across the tick genera [32, 86]The high prevalence, vertical transmission, and B-vitamin metabolic provisioning role of *Francisella*-like endosymbionts in ticks provides robust evidence for their specialized, obligate niche, which supports the interpretation that they are topologically independent of the general commensal community’s network dynamics. It has a single dominant ASV, “Francisella_ASV7”, which completely saturates the bacteriome of *H. lusitanicum*, where it accounts for ~ 90% of all reads. It is definitively a “core dominant” specialist for that species with *Midichloria* acting as the clear secondary dominant (~ 6%) to constitute *Francisella*-*Midichloria* signature that defines the bacteriome of this tick species.

Finally, our analysis suggests that global network stability is driven by association patterns reflecting cooperation rather than competition. The “Central Connectors” that maintain network integrity—most notably *Sphingomonas* as a universal inter-species connector, alongside species-specific modular drivers like *Roseomonas* (Table 3), exhibited an interaction weight balance of > 98% positive links (see Figs. 8 and 9). We propose that these Connectors act as “potential topological stabilizers”. Their central position is hypothesized to stem from the production or processing of key shared metabolites (e.g., products of heme digestion or nitrogen metabolism) that could benefit multiple, taxonomically distant microbial modules. This finding has significant translational implications: targeting these Central Facilitator Connectors represents a promising theoretical strategy for microbiome disruption. Unlike targeting abundant but isolated endosymbionts, disrupting these central “inferred structural nodes”, which represent potential topological pathways through which the majority of cooperative metabolic exchanges are predicted to occur, would likely destabilize the entire microbial network, potentially impacting tick fitness and reproductive success [40].

## Conclusions

The analysis of the bacteriome, rigorously conducted at Amplicon Sequence Variant (ASV) resolution, established that the tick microbiome is a consistently highly modular and hierarchical ecosystem. Significant differences in composition, driven primarily by *Francisella*, *C. Midichloria*, and *Coxiella*, confirmed a strong species-specific influence. Despite this variation, the study identified a conserved seven-genus core microbiome, including key taxa such as *Coxiella*, *Acinetobacter*, and *Francisella*, that is shared across the five most prevalent tick species.

This highly modular architecture provides critical ecological resilience, which is structurally reinforced by the topological role of the obligate endosymbionts. ASVs corresponding to *Coxiella*, *Rickettsia*, and *Francisella* were universally classified as Peripheral or simple Connector nodes, regardless of the stringent PT applied. This topological finding confirms their functional isolation from the global network dynamics, suggesting their vital roles in vertical transmission and nutrient provisioning operate independently of the complex co-occurrence patterns exhibited by the resident commensal microbiota.

Network stability and connectivity within the tick microbiome were found to be maintained by an influential minority of ASVs (1% to 4% of the total network). The network’s architectural integrity is rooted in the fact that critical control elements, comprising both universal connectors (global flow) and species-specific drivers (local cohesion), rely overwhelmingly on positive interactions, maintaining an average of 98.7% facilitative links (Table 3). This near-exclusive reliance on positive correlations is strongly supported by the confirmed mutualistic relationships established in ticks, particularly the dependency on metabolic pathways provided by dominant obligate symbionts like *Coxiella* (Table 3). This mechanism explains why ASVs holding critical network positions function as central facilitators; they are likely key contributors to or recipients of shared metabolic pools. This stability is driven by specific keystone taxa, recurrently including *Sphingomonas* as a cross-species connector and *Roseomonas* as a localized driver, demonstrating that statistically drive the network’s topological organization is achieved through ecological commensalism, likely via metabolic support, rather than competitive suppression. The identification of these cooperative structural connectors represents an actionable finding for vector control, as these nodes constitute the vulnerable topological chokepoints of the tick microbiome. Consequently, intervention strategies should shift from targeting resilient obligate symbionts toward the selective disruption of these central facilitators, whose removal would likely trigger a network-wide disconnect, destabilizing the microbial ecosystem and potentially impacting tick fitness and pathogen competence.

The pronounced interspecific variation in topological stability highlights fundamental differences in how network control is mediated and maintained in the tick bacteriome (Table 3). The reliance of *Rhipicephalus bursa* on weak, peripheral correlations, which completely collapsed its local driver structure under stricter thresholds, suggests that its perceived connectivity is highly fragile and vulnerable to symbiont saturation. Conversely, the unwavering stability of *D. marginatus* and *H. sulcata*, which operate under robust specific drivers (such as *Roseomonas*, *Klenkia*, and *Acinetobacter*), indicates that a high-strength, resilient core is vital to support complex, integrated network topologies. Thus, structural resilience is not uniform across tick species but is directly correlated with the presence of diverse topological drivers capable of securing connectivity using strong, cooperative ecological links.

Given the near-total suppression of negative interactions, it is highly probable that these critical nodes maintain their position of control by facilitating ecological cooperation, likely through the production and sharing of essential metabolites or by occupying non-overlapping ecological niches, rather than through competitive exclusion or suppression.

## Supplementary Information

Below is the link to the electronic supplementary material.

Supplementary Material 1 Interactive 3D. Reconstruction of the *D. marginatus* nest. The model visualizes the spatial architecture and internal volume of the structure. The interactive rendering allows for 360-degree rotation along the three spatial axes (X, Y, and Z) to examine the nest topology and entrance orientation (HTML 882 KB)

Supplementary Material 2 Interactive 3D. Reconstruction of the *R. bursa *nest. The model visualizes the spatial architecture and internal volume of the structure (HTML 895 KB)

## Data Availability

The raw sequencing data are currently pending assignment of a BioProject accession number, which will be provided prior to publication. All custom code, scripts, and processed data matrices required to reproduce the analyses and figures presented in this manuscript are freely available on GitHub at: [https://github.com/jmarquez20/Tick\_microbiome\_Carzorla\_2026](https:/github.com/jmarquez20/Tick_microbiome_Carzorla_2026). Data are available from the authors upon reasonable request.
