# Supplementary material for: Beyond Dominant Symbionts: Low-Abundance Taxa Govern Microbial Network Topology in Sympatric Ticks
Source: Microb Ecol. 2026 Jul 20;89:189. doi: 10.1007/s00248-026-02842-y (PMC13672775; doi:10.1007/s00248-026-02842-y)
Supplement: Supplementary file 1 [file 248_2026_2842_MOESM1_ESM.html]

scatterplotThree
